# Missing Clinical Information in NHS hospital outpatient clinics: prevalence, causes and effects on patient care

**DOI:** 10.1186/1472-6963-11-114

**Published:** 2011-05-23

**Authors:** Susan J Burnett, Vashist Deelchand, Bryony Dean Franklin, Krishna Moorthy, Charles Vincent

**Affiliations:** 1 Centre for Patient Safety and Service Quality (CPSSQ), Imperial College London, Department of Surgery, Division of Surgery and Cancer, Faculty of Medicine, Room 508 Medical School Building, St Mary's Campus, Norfolk Place, London, W2 1PG, UK; 2 CPSSQ, Imperial College London, Department of Surgery, Division of Surgery and Cancer, Faculty of Medicine, 5th Floor Medical School Building, St Mary's Campus, Norfolk Place, London, W2 1PG, UK; 3 Centre for Medication Safety and Service Quality, The School of Pharmacy, University of London and Imperial College Healthcare NHS Trust, Pharmacy Department, Charing Cross Hospital, London, W6 8RF, UK; 4 Department of Surgery, Division of Surgery and Cancer, Academic Surgical Unit, Imperial College London, 10th Floor, QEQM Wing, St Mary's Hospital, London W2 1NY, UK; 5 CPSSQ, Imperial College London, Department of Surgery, Division of Surgery and Cancer, Faculty of Medicine, 10th Floor QEQM Wing, St Mary's Hospital, London, W2 1NY, UK

## Abstract

**Background:**

In Britain over 39,000 reports were received by the National Patient Safety Agency relating to failures in documentation in 2007 and the UK Health Services Journal estimated in 2008 that over a million hospital outpatient visits each year might take place without the full record available. Despite these high numbers, the impact of missing clinical information has not been investigated for hospital outpatients in the UK.

Studies in primary care in the USA have found 13.6% of patient consultations have missing clinical information, with this adversely affecting care in about half of cases, and in Australia 1.8% of medical errors were found to be due to the unavailability of clinical information.

Our objectives were to assess the frequency, nature and potential impact on patient care of missing clinical information in NHS hospital outpatients and to assess the principal causes. This is the first study to present such figures for the UK and the first to look at how clinicians respond, including the associated impact on patient care.

**Methods:**

Prospective descriptive study of missing information reported by surgeons, supplemented by interviews on the causes.

Data were collected by surgeons in general, gastrointestinal, colorectal and vascular surgical clinics in three teaching hospitals across the UK for over a thousand outpatient appointments. Fifteen interviews were conducted with those involved in collating clinical information for these clinics.

The study had ethics approval (Hammersmith and Queen Charlotte's & Chelsea Research Ethics Committee), reference number (09/H0707/27). Participants involved in the interviews signed a consent form and were offered the opportunity to review and agree the transcript of their interview before analysis. No patients were involved in this research.

**Results:**

In 15% of outpatient consultations key items of clinical information were missing. Of these patients, 32% experienced a delay or disruption to their care and 20% had a risk of harm. In over half of cases the doctor relied on the patient for the information, making a clinical decision despite the information being missing in 20% of cases. Hospital mergers, temporary staff and non-integrated IT systems were contributing factors.

**Conclusions:**

If these findings are replicated across the NHS then almost 10 million outpatients are seen each year without key clinical information, creating over a million unnecessary appointments, and putting nearly 2 million patients at risk of harm. There is a need for a systematic, regular audit of the prevalence of missing clinical information. Only then will we know the impact on clinical decision making and patient care of new technology, service reorganisations and, crucially given the present financial climate, temporary or reduced staffing levels. Further research is needed to assess the relationship between missing clinical information and diagnostic errors; to examine the issue in primary care; and to consider the patients perspective.

## Background

Clinicians report informally that missing clinical information is a daily occurrence and a constant source of irritation and delay. The problem appears to be so endemic as to be almost invisible with clinicians finding ways to work around missing information, making decisions as best they can with assistance from patients, who endeavour to make up for the deficiencies in the healthcare system. Although the problem is well known amongst clinicians, surprisingly little is known about its true scale and, more importantly, its actual impact on patient care.

Primary care has been more extensively studied than hospital care. For instance Smith and colleagues at the University of Colorado Health Sciences Centre [1] found that 13.6% of patients' consultations in primary care in the United States had missing clinical information and that this adversely affected patient care in about half of cases. Missing clinical information has been found to be a contributory factor in medical errors. For instance, Dovey et al [2] in examining 344 reports of medical errors from US family physicians, found that 7.8% were due to the unavailability of information that should have been in the patient's medical records. In this study the majority of error reports were due to administrative problems with 44% of all error reports perceived by the physician to be associated with adverse consequences for the patient or family. Wilson et al [3] found that 1.8% of 2353 adverse events from the Quality in Australian Healthcare Study were due to 'acting on insufficient information' with 26.4% of these leading to permanent disability.

In Britain over 39,000 reports were received by the National Patient Safety Agency relating to failures in documentation in 2007 [4]. The Health Services Journal estimated in 2008 [5] that 54,000 of over two million outpatient appointments in 49 hospitals between 2006 and 2008 took place without the patient's full records and that over a million outpatient visits each year might take place without the full record available. However the survey used hospitals' own estimates of missing information; those who carried out a full audit reported rates 11 times higher than those who simply provide estimates (3.3% versus 0.3%). Despite these high numbers, the impact of missing clinical information has not been reported in the UK, for example we do not know how clinicians proceed in the absence of key information nor how missing information impacts on patient care.

The aim of the present study was to assess the prevalence, nature and impact of missing information in NHS hospital outpatient appointments and to make an initial assessment of the principal causes of missing information. This study formed part of a wider examination of the reliability of clinical systems including studies of handover, medication delivery, systems for intravenous line insertion and the availability of equipment in the operating theatre.

## Methods

### Design

Prospective descriptive study of missing information reported by surgeons supplemented by a series of interviews exploring the causes of missing information

### Setting

The study was conducted in general, gastrointestinal, colorectal and vascular surgical clinics in three large teaching hospitals, A, E and G across the UK, details of the selection process are available in the full research report from the authors. All had paper-based medical records but each organisation had some test results and other information available on computer. A process map was produced of the steps involved in assembling paper and electronic information for clinics in general in each hospital. These were found to be broadly similar, the main difference between organisations being what information was available via computer, whether this was available in one place or required different logins, and the availability of computers in clinics to access this information.

### Assessment of missing information

Working initially with surgeons in site G and then those in A and E, a core data set was agreed for clinical information that should be available to the surgeon during a typical outpatient appointment if needed, including new and follow up patients, both pre- and post- operatively as follows:

• Past medical history

• Referral letter/other specialty letter

• Discharge summary

• Current medication

• Allergies

• Radiology/imaging results

• Diagnostic test results

• Procedure notes/anaesthetic record

• Electrocardiogram (ECG) report

• Blood laboratory results

• Outpatient medical records/last clinic letter

A form was designed for surgeons to complete for each patient where either the whole medical record or the identified information was judged by them to be missing. The doctors were asked to record details of:

• What information was required, but missing (test results, images, referral letter etc)

• Whether or not they relied on the patient for any of the clinical information that was missing

• Whether they made a clinical decision without the information

• Whether or not the patient required another appointment because the information was missing

• The impact on patient care (delay in management, cancellation of procedure etc) as judged by the doctor using a four point scale (none, minor, moderate, severe)

• The potential risk of harm to the patient as judged by the doctor using a five point scale (no threat, minor, moderate, potential adverse event, potential serious adverse event)

All patients attending the clinics, both pre-and post-operative, were included in the study and data collected for each patient. At site A, a researcher distributed and collected the forms at each clinic. At site E this was done by the clinic nurses and at site G by a medical records supervisor.

### Sample

Our sample size was based on a primary binomial outcome of whether or not all of the required information was available. A target of 400 patients in each organisation was agreed. This enabled the observed proportion across the whole sample to be estimated to the nearest 0.05 (in the worst case) at a 95% confidence level, recognising that the subsets within the sample will have wider confidence intervals. The total number of patients attending each clinic was obtained from the clinic records. Data were collected between July and September 2009.

### Interviews to explore the causes of missing information

Reasons for missing information were then explored through interviews with key staff. Participants were eligible to be interviewed if they were involved in the delivery or use of clinical information in the outpatient setting. The local collaborator in each organisation suggested eligible staff and invited them to participate.

A semi-structured interview guide was used and all interviews were conducted by the same interviewer exploring the likely causes of information not being available when needed and any recommendations for improving the reliability of these systems. Interviews were conducted between September and November 2009 and were of 20 to 30 minutes' duration. With interviewees' consent, interviews were recorded and transcribed verbatim. We performed qualitative analysis with the aid of NVIVO (version 8). Framework analysis was undertaken using the accident causation model (Table 1)[6]. Associated sub-themes were then drawn from this data. Comparison and refinement was carried out between two researchers.

**Table 1:** Factors in the Accident Causation Model [6]

1. Institutional context	• Economic and regulatory context
2. Organisational and management factors	• Financial resources and constraints
	• Organisational structure
	• Policy standards and goals
	• Safety culture and priorities

3. Work environment	•Staffing levels and skills mix • Workload and shift patterns • Design, availability, and maintenance of equipment • Administrative and managerial support

4. Team factors	• Verbal communication • Written communication • Supervision and seeking help • Team structure

5. Individual (staff) factors	• Knowledge and skills • Motivation Physical and mental health

6. Task factors	• Task design and clarity of structure • Availability and use of protocols • Availability and accuracy of test results

7. Patient characteristics	• Condition (complexity and seriousness) • Language and communication • Personality and social factors

## Results

### The prevalence of missing information in surgical outpatient clinics

From the total sample of 1,161 patients across three organisations, 18 (1.5%) had their entire medical record missing and 175 (15%) had one or more pieces of clinical information missing (Table 2). When information was missing, the average number of items missing per patient was 1.8 in organisation A; 1.7 in organisation E; and 2.4 in organisation G.

**Table 2:** Prevalence of missing information in surgical outpatient appointments

Organisation	Total number of patients in the sample	Records unavailable (percentage of all patients)	Confidence intervals	Number of patients with missing information (percentage of all patients)	Confidence intervals
A	411	1 (0.2%)	0 to 0.7%	18 (4%)	2.4% to 6.4%

E	423	3 (0.7%)	0 to 1.5%	113 (27%)	22.5% to 31%

G	327	14 (4.3%)	2.1% to 6.5%	44 (13%)	9.8% to 17.1%

TOTAL	1161	18 (1.5%)	0.8% to 2.3%	175 (15%)	13% to 17.1%

### Type of missing information

**Figure 1:**
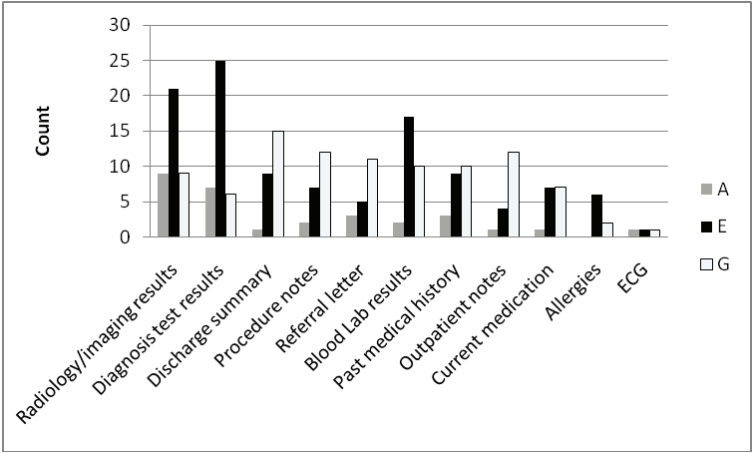
**Count of missing information by type in each organisation**.

The type of information missing varied between organisations. Radiological/imaging, diagnostic and blood test results were missing most frequently overall and in particular in organisation E (Figure 1). In organisation G written communication was missing more frequently (discharge summary, procedure notes, referral letters).

### Impact on patient care

The doctors perceived there to have been an impact on patient care, such as delays in patient management, cancellation of operation etc in 55 patients (4.7% of the whole sample, or 32% of those with missing information). Twenty patients were given a second appointment because of missing information (1.7% of all patients in the sample). Doctors reported making a clinical decision without key information in 37 patients (21% of those with missing information). When a doctor made a clinical decision without key information, the most common type of information missing was diagnostic test results and blood laboratory results. Overall when information was missing the doctors relied on patients for help with the missing information on more than half of all occasions (58%).

In 35 patients (3% of the whole sample; 20.5% of those with missing information), doctors perceived there to be a risk of harm associated with the missing information. This ranged from a minor threat (n = 22) to risk of a serious adverse event (n = 1). There were three cases where the impact on patient care was deemed to be severe and here the following specific items of information were missing: diagnostic test results (n = 1); both radiology/imaging results and diagnostic test results (n = 1); and procedure notes (n = 1).

### System failures analysis

Fifteen people were interviewed, five from each of the three organisations, including surgeons, medical records staff, medical secretaries, outpatient nurses and key staff in radiology and pathology. Apart from the surgeons, the interviewees were asked about the systems as they applied to outpatient clinics in general. Table 3 sets out the quotes from participants. The following themes emerged as being of particular importance.

**Table 3:** quotes from participants

Quote reference	Quote and participant reference
1	*"...where the patient needs either a biopsy, maybe they need some form of staging scan, may need something else, and frankly it, you want everything lined up in the right order, and organisationally, that can be logistically very difficult." Surgeon, organisation E*

2	*" Computers aren't in every room ... we have a computer in the nurses' station, which is quite a busy area just with you trying to get results" Outpatient nurse, organisation E*

3	*"I have to say one thing is operating notes. from previous operations are sometimes very important and I find it hard in this hospital sometimes to find these. And the problem is maybe I don't know where they are but I have the feeling they are handwritten notes" Surgeon, organisation A*

4	*"Some of the doctors forget their password or they haven't got a password so they can't always access the system... the x-ray system it's a different password" Outpatient nurse, organisation E*

• The difficulties of aligning a patient's complex pathway ensuring that all tests are completed and reported before they return for their follow up appointment (Table 3 quote 1).

• Across all organisations the problems of finding paper based medical records at short notice was described as a particular problem for urgent appointments, re-bookings and waiting list initiative clinics. This was exacerbated by the proliferation of unstandardised forms for the ordering of different types of test.

• Hospital mergers have brought a number of organisational problems including patients having multiple hospital numbers.

• Problems with paper systems were wide ranging including poor storage facilities in offices and clinics meaning medical records were stored on the floor, to problems with medical records tracking. Poorly fixed folders were cited by staff in two organisations with 'fat folders' for sick patients often falling apart.

• Problems with computer systems included the design of the software, the age and availability of terminals, problems with passwords and logins required to access multiple systems. Old technology meant that some surgeons could not view electronic images from scans and X-rays during an appointment because they took too long to load. Some clinics did not have enough terminals for the doctors to access information during the clinic (Table 3 quote 2).

• Running both paper and computer systems in parallel was given as a problem in all three organisations with staff not knowing where to look for information (Table 3 quote 3).

• In all three organisations problems were experienced with temporary staff or staff covering shifts and being unfamiliar with the local systems. This led to problems with them being unable to find information when required, not having access to certain computer systems or filling in forms wrongly (Table 3 quote 4).

### Limitations

Our data relied on collection by busy doctors in outpatient clinics and may therefore be subject to under-reporting. We also relied on doctors to assess their perceptions of risk to patients and the impact of missing information on the patient's care using a simple scoring system. This type of perception scoring is useful in gaining an assessment of the issue in question but perceptions of risks are likely to vary between clinicians and the results should be seen in this light. There is also the potential for bias if clinicians are frustrated with the system and exaggerated the problems in order to highlight this to managers. The organisations selected were all large teaching hospitals and more research is needed to confirm whether the size or type of hospital affects the availability of clinical information, and whether the same kinds of problems are apparent in different kinds of clinic.

### Extrapolation of the findings across the United Kingdom the results for the UK

Despite the limitations of this study, we can make some tentative estimates of the potential scale of the problem of missing information in outpatient appointments on the basis that (a) the systems for getting information to clinics applied generally to clinics in the hospitals; (b) these systems involved assembling paper and electronic information for a patient visit with steps that all hospitals would have to undertake regardless of size; (c) our estimates of missing records in NHS outpatient clinics are in line with those found previously in the UK[5]. Table 4 has been produced based on figures taken from the NHS Information Centre, ISD Scotland and Stats Wales.

**Table 4:** Extrapolation of study findings to the entire NHS

Percentages found in this study	Estimated annual numbers of patients if study findings are applied to:	
	**General surgery outpatients** **(n = 4.4 million)**	**All outpatient attendances** **(n = 66 million)**

1.5% missing medical records	66,300	991,300

15% missing clinical information	663,000	9,913,000

4.7% impact on patient care	207,700	3,106,000

1.7% new appointment booked	75,150	1,124,000

3.2% decision without information	141,500	2,115,000

3% risk of harm	132,600	1,983,000

## Discussion

This is the first study to present figures on missing clinical information in outpatient clinics in the UK across organisations, and the first to look at how clinicians respond including the associated impact on patient care. Clinicians in our study were faced with making decisions about patient care without key items of clinical information in 15% of 1,161 patients seen in the outpatient clinics studied. Of those patients with missing clinical information, 32% experienced a delay or disruption to their care, 20% had a risk of harm. In over half of cases the doctor relied on the patient for the information, making a clinical decision despite the information being missing in 20% of cases. Hospital mergers, temporary staff and non-integrated IT systems all contributed to the problems of missing clinical information.

The prevalence of missing entire medical records was relatively low in the clinics studied, nevertheless we found ten times more patients being seen with one or more pieces of important clinical information missing. NHS organisations are encouraged to monitor missing medical records as part of the national information governance requirements [7] but to date have not been asked to collect information about what is missing from these records. Each time important clinical information is missing our results show that there is opportunity for the patients care to be delayed and also for patient harm. This is clearly an important issue both for patient satisfaction and for patient safety that to date has not been measured across the NHS.

The electronic medical record, if fully introduced, should considerably reduce missing clinical information and potentially also achieve greater integration between primary and secondary care. However it may not resolve all the problems noted here. Patients with two or more hospital numbers for instance may have more problems with an electronic system, and demands on locum staff may be greater with increased chance of incorrectly entered information. We believe that if the impact of an electronic record is to be fully understood it should be preceded by a substantial study of the nature and prevalence of missing information which can be tracked over time as the electronic systems are introduced particularly as any electronic system will undoubtedly bring new risks as well as new benefits[8]. We therefore recommend that a method for auditing the prevalence of missing clinical information is developed across the NHS and is measured systematically and regularly to monitor improvements. Only then will we know the impact on clinical decision making and patient care of new technology, service reorganisations and, crucially given the present financial climate, temporary or reduced staffing levels. Further research is needed to assess the impact of missing clinical information on diagnostic errors and in primary care, to assess the prevalence of missing clinical information and to consider the impact in primary care of missing information in the hospital. Work is also needed to understand the patient's perspective, for example should hospitals postpone appointments until all the necessary information is available, or give patients the choice of whether to be seen with no records?

## Conclusions

Finally we would emphasise that the problem of missing information may not be quite as intractable as it sometimes seems and that current systems and processes could probably be dramatically improved even in the absence of electronic systems. The most striking finding from our study was that while all three organisations had problems, they were not the same problems. In each hospital, some systems worked well and others poorly. This suggests that it is possible for all the existing systems to run more effectively if missing information was given higher priority and if sufficient effort was given to applying the standard armament of process improvement tools to the problem[9]. We recommend that those with systems wide management responsibilities receive training in systems theory and practice. Improving the reliability of clinical records systems would make care safer for patients, less frustrating for clinicians, reduce delay and duplication and save a great deal of money by avoiding extra appointments.

## Competing interests Declaration

The authors declare that they have no competing interests.

## Authors' contributions

SJB was the lead for the research topic, analysed the data and prepared the research report. VD collected data, conducted interviews, analysed qualitative data into themes. BDF was the Principal Investigator for overall study of reliability in clinical systems. KM provided clinical advice and advised on research methods. CV was advisor to the research project. All authors read and approved the final manuscript.

## Pre-publication history

The pre-publication history for this paper can be accessed here:

http://www.biomedcentral.com/1472-6963/11/114/prepub
